# Perinatal outcomes from preterm and early term births in a multicenter cohort of low risk nulliparous women

**DOI:** 10.1038/s41598-020-65022-z

**Published:** 2020-05-22

**Authors:** Renato T. Souza, Maria L. Costa, Jussara Mayrink, Francisco E. Feitosa, Edilberto A. Rocha Filho, Débora F. Leite, Janete Vettorazzi, Iracema M. Calderon, Maria H. Sousa, Renato Passini, Philip N. Baker, Louise Kenny, Jose G. Cecatti, The Preterm SAMBA study group*, Mary A. Parpinelli, Karayna G. Fernandes, Rafael B. Galvão, José Paulo Guida, Danielly S. Santana, Daisy de Lucena, Benedita Sousa, Elias F. Melo, Danilo Anacleto, Lucia Pfitscher, Luiza Brust, Bianca F. Cassettari, Kleber G. Franchini, Rodolfo C. Pacagnella

**Affiliations:** 1 Department of Obstetrics and Gynaecology, University of Campinas (UNICAMP) School of Medical Sciences, Campinas, SP Brazil; 2 MEAC – School Maternity of the Federal University of Ceará, in Fortaleza, CE Brazil; 3 Department of Maternal and Child Health, Maternity of Clinic Hospital, Federal University of Pernambuco, in Recife, PE Brazil; 4 Department of Obstetrics and Gynaecology, Maternity of the Clinic Hospital, Federal University of RS, Porto Alegre, RS Brazil; 5 Department of Obstetrics and Gynaecology, Botucatu Medical School, Unesp, Botucatu, SP Brazil; 6 Statistics Unit, Jundiai School of Medicine, Jundiaí, SP Brazil; 7 College of Life Sciences, University of Leicester, Leicester, United Kingdom; 8 Faculty of Health and Life Sciences, University of Liverpool, Liverpool, UK; 9 LNBio - Brazilian Biosciences National Laboratory and School of Medical Sciences, University of Campinas (UNICAMP), Campinas, SP Brazil

**Keywords:** Medical research, Epidemiology

## Abstract

Preterm birth is the major contributor for neonatal and under-five years mortality rates and also accounts for a short- and long-term adverse consequences up to adulthood. Perinatal outcomes may vary according to lots of factors as preterm subtype, late prematurity, which account for the vast majority of cases, country and population characteristics. An under-recognition of the perinatal outcomes and its associated factors might have underpowered strategies to provide adequate care and prevent its occurrence. We aim to estimate the frequency of maternal and perinatal outcomes in women with different categories of preterm and term births, factors associated with poorer perinatal outcomes and related management interventions. A multicentre prospective cohort in five maternities in Brazil between 2015 and 2018. Nulliparous low-risk women with singletons were included. Comprehensive data were collected during three antenatal visits (at 19–21weeks, 27–29 weeks and 37–39 weeks). Maternal and perinatal outcomes were also collected according to maternal and neonatal medical records. Women who had spontaneous (sPTB) and provider-initiated (pi-PTB) preterm birth were compared to those who had term birth. Also, late preterm birth (after 34 weeks), and early term (37–38 weeks) were compared to full term birth (39–40 weeks). Bivariate analysis estimated risk ratios for maternal and adverse outcomes. Finally, a multivariate analysis was conducted to address factors independently associated with any adverse perinatal outcome (APO). In total, 1,165 women had outcome data available, from which 6.7% had sPTB, 4.0% had pi-PTB and 89.3% had a term birth. sPTB and pi-PTb were associated with poorer perinatal outcomes, as well as late sPTB, late pi-PTB and early term neonates. pi-PTB (RR_adj_ 8.12, 95% CI [2.54–25.93], p-value 0.007), maternal weight gain between 20 and 27 weeks <p10 (RR_adj_ 2.04, 95% CI [1.23–3.38], p-value 0.018) and participants from the Northeast centres (RR_adj_ 2.35, 95% CI [1.11–4.95], p-value 0.034) were independently associated with APO. According to our findings, Brazil would benefit from strategies to more accurately identify women at higher risk for PTB, to promote evidenced-based decision in preterm and early term provider-initiated deliveries, and to prevent perinatal adverse outcomes.

## Introduction

Preterm birth (PTB) is associated with short- and long-term adverse outcomes for the neonate. In addition, it is the leading cause of neonatal death and also a contributor to the under-five mortality rate ^1–3^. A secondary analysis of a World Health Organization study evaluating almost 300,000 deliveries in 29 countries showed that perinatal outcomes as stillbirth and early neonatal deaths vary according to the preterm birth subtypes^4^. The rates are approximately 30% lower in spontaneous than in provider-initiated preterm birth.

Preterm birth subtype can be classified according to its motivation. Spontaneous preterm birth (sPTB) is defined as any preterm birth occurred due to spontaneous onset of labour or premature rupture of membranes (PROM) and provider-initiated preterm birth (pi-PTB) when preterm birth was indicated by health care providers due to maternal and/or fetal conditions^1^. It can be also divided according to gestational age at delivery in extremely preterm (<28 weeks), very preterm (28–31 weeks), moderate preterm (32–33 weeks), and late preterm (34–36 weeks)^1,5^. Not only preterm, but also early term deliveries (37–38 weeks) are associated with adverse perinatal outcomes^6–8^. Both groups are close to 37 weeks and, because of that, related outcomes are usually underestimated, especially in provider-initiated deliveries. Neonatal and infant mortality rates are around 2 times higher in these groups compared to 39 weeks neonates^8^.

Secondary analyses of the Birth in Brazil study, a hospital-based cross-sectional study that included women from 266 hospitals from February 2011 to October 2012, confirm that PTB is the leading cause of neonatal mortality in Brazil^9^. The factors associated with higher rates of neonatal mortality included peregrination, not using a partograph, delivering before 32 weeks and delivering at a unit of the public unified health system^9^. Less than 20% and 15% of the public and private maternities have an intensive care unit, respectively^10^. In addition, 67.1% in the public and 16.5% in private maternities have ambulances for neonates. Preterm birth, then, has a great burden to the Brazilian health system, where about 3 million births occurs annually and around 300,000 are preterm^11^.

Determining the frequency of perinatal and neonatal outcomes is important for allocating human and infrastructure resources to properly provide care for preterm neonates and infants and planning adequate strategies to monitor PTB preventive interventions. Therefore, the perinatal outcomes related to preterm birth and to the different PTB subtypes are of great interest, especially in countries like Brazil where it represents a great burden to the health system. In the current study, we aim to estimate the frequency of maternal and perinatal outcomes in women with different categories of preterm and term births, factors associated with poorer perinatal outcomes and related management interventions.

## Methods

We conducted a longitudinal multicentre cohort study in five referral obstetric centres in Brazil between July 2015 and July 2018, called Preterm SAMBA. The research protocol, methodological procedures, including the selection of the participating centres, and others aspects of the study implementation and progress had already been detailed^12,13^. In brief, the Preterm birth cohort was comprised of low-risk nulliparous women with singleton pregnancies. Exclusion criteria were repeated abortions (≥3), fetal major malformation, chronic hypertension (using antihypertensive drug or if moderate hypertension), diabetes type I or II, renal disease, HIV, sickle cell disease, uterine anomalies, history of cervical knife cone procedures, use of steroids (≥3 months), aspirin, calcium, fish oil, vitamin C or E or heparin. Participants were enrolled at 19–21 weeks of gestation. Then, antenatal visits were performed at 19–21 weeks, 27–29 weeks and 37–39 weeks. Data regarding sociodemographic characteristics, maternal and family medical history, habits, anthropometric measures, height, pregnancy characteristics, occurrence of complications and other maternal and fetal outcomes were collected and entered in an online database during the three study visits. Childbirth and postpartum data were retrospectively collected by reviewing maternal and neonatal medical records until discharge. Maternal weight gain rate per week (WGR) was calculated according to the difference of weight between the first two visits (19–21 weeks and 27–29 weeks).

The study was approved by the Ethical Review Board of each participant centre and was endorsed by the Brazilian National Committee for Ethics in Research (CONEP). The study complies with the 1989 Declaration of Helsinki and the Brazilian national regulations for studies in human beings stated by the National Health Council (Resolution CNS 466/12). All participating women signed an informed consent form before enrolment.

### Outcomes and variables

Preterm birth was defined as any birth occurred before 37 weeks of gestation. A term birth group was comprised of all women who had birth ≥37 weeks of gestation. Then, three groups were established according to gestational age and preterm birth subtypes. Spontaneous preterm birth (sPTB) included women who had preterm birth due to spontaneous onset of labour or premature rupture of membranes. Provider-initiated preterm birth (pi-PTB) was defined as a preterm birth due to medical indication on account of maternal or fetal conditions/complications. The mode of delivery does not play a role in the definition of the PTB subtype. So, if women had a pPROM and, then, an (suspected or confirmed) intra-uterine infection, it would be classified as spontaneous preterm birth. Although a medical decision was made, the primary complication and driver was pPROM. In addition, neonatal outcomes from late sPTB, late pi-PTB (34–36 weeks) and early term (37–38 weeks) and post term (41–42 weeks) cases were compared to full term cases (39–40 weeks).

Maternal characteristics (Region site; ethnicity; annual family income; source of prenatal care; smoking status; previous maternal condition; cervical length from 18 to 24 weeks <25 mm; weight gain rate per week 20–27 weeks <Q1; weight gain rate per week 20–27 weeks <Q2; weight gain rate per week 20–27 weeks <p10; weight gain rate per week 20–27 weeks >p90), preterm subtypes (sPTB; pi-PTB), gestational age at birth, and pregnancy complications such as preeclampsia and Hyperglycaemia in pregnancy were addressed as factors (exposures) potentially associated with adverse perinatal outcomes. Maternal weight gain rate per week (WGR) was calculated according to the difference of weight between the first two visits (19–21 weeks and 27–29 weeks).

Maternal and neonatal outcomes included: onset of labour and mode of delivery; hyperglycaemia in pregnancy (HIP), defined by an initial fasting plasma glucose ≥92 mg/dL or altered 75 g oral glucose tolerance test performed between 24 and 28 weeks of gestation that means fasting plasma glucose ≥92 mg/dL or 1h-postglucose load ≥180 mg/dL or 2h-postglucose load ≥153 mg/dL; preeclampsia, defined as having systolic blood pressure ≥140 or systolic blood pressure ≥90 mmHg after 20 weeks gestation on at least two occasions apart of 20 min, and/or proteinuria (24-h urinary protein ≥300 mg or urine dipstick ≥++) and/or severe maternal complications; Apgar score <7 at 5 minutes; fetal malformation diagnosed/confirmed after birth; need of intubation after birth; neonatal sepsis (confirmed or suspected); adequacy of birthweight according to GROW customized birthweight centiles^14^; phototherapy for jaundice; neonatal intensive care admission; length of maternal and NICU stay. A composite outcome “any adverse perinatal outcome” (APO) was operationally defined as one of the following adverse neonatal outcomes: Apgar score <7 at 5 minutes, fetal or neonatal death, intubation at birth, birth asphyxia (according to medical records following the institutional criteria which included 5 minute Apgar <7, umbilical cord blood pH < 7.0 within 1-hour after birth, base excess >10, failure to initiate spontaneous and sustained breathing after 10 minutes of resuscitation or clinical signs of neonatal neurologic dysfunction related to perinatal asphyxia such as seizures, hypoxic ischemic encephalopathy, tone abnormalities or multi-organ involvement such as kidney, lung, liver and intestine, NICU stay >7 days, neonatal sepsis (early or late, suspected or confirmed - suspected sepsis was considered when the newborn had organ dysfunction caused by infection and treated without confirmation and confirmed sepsis was considered when infection newborn was treated of a confirmed infection by positive urine, blood, liquor or other culture or PCR), cyanosis, hypoglycemia (plasma glucose less than 50 mg/ or requiring intravenous bolus of dextrose), respiratory distress or mechanical ventilation, discharge home on oxygen. Antenatal and peripartum management characteristics were also addressed, including vaginal progesterone (any dose), cerclage, pessary, steroids and tocolysis use. For mode of delivery, elective C-section was considered when it was indicated in women without labour, and also for women who failed induction (did not initiated labour). Intrapartum C-section were considered when C-section was performed in women during labour, including women at any stage of labour following induction.

### Statistical analysis

Maternal characteristics were compared between the sPTB, pi-PTB and term groups using chi-squared test (χ^2^). Only p-values < 0.05 were considered statistically significant. Bivariate analyses by Poisson logistic regression were carried out to calculate the risk ratios and 95% CI for maternal and perinatal outcomes, including pregnancy management characteristics in sPTB, pi-PTB compared to term and also to late sPTB, late pi-PTB, and early term compared to full term. A multivariate analysis by Poisson logistic regression was conducted to address factors independently associated with any adverse perinatal outcome (APO). The sample size calculation for the had been described elsewhere and it was based on the minimum population required to assess metabolomics markers as predictors for sPTB^12^.

We used Stata v. 7.0 (StataCorp) and SPSS v. 20.0 (IBM SPSS Statistics, USA) to perform all statistical analysis. All the analyses (p-values and 95% CI of the RR) accounted for the primary sampling unit to account for cluster-design effect, using the variance estimator based on the first-order Taylor series linear approximation. *Post-hoc* analysis: we did not apply any statistical method to deal with missing data, considering that only one variable had missing values more than 10%. The number of missing information was provided in the table’s footnotes. The variable with the highest proportion of missing values was HIP, which missing represented 13.5% of included cases. The second with the higher proportion of missing was Apgar score (5.6%). All others had lower frequencies of missing values.

### Ethics approval and consent to participate

The current study was approved by each local Institutional Review Board (IRB) and amended by the Brazilian National Committee for Ethics in Research (CONEP) - Letter of approval 1.048.565 issued on 28th April 2015. The study complies with national and international regulations for experiments in human beings, including the resolution CNS 466/12 of the Brazilian National Heath Council and the 1989 Declaration of Helsinki. All women signed an informed consent form before enrolment.

## Results

**Figure 1:**
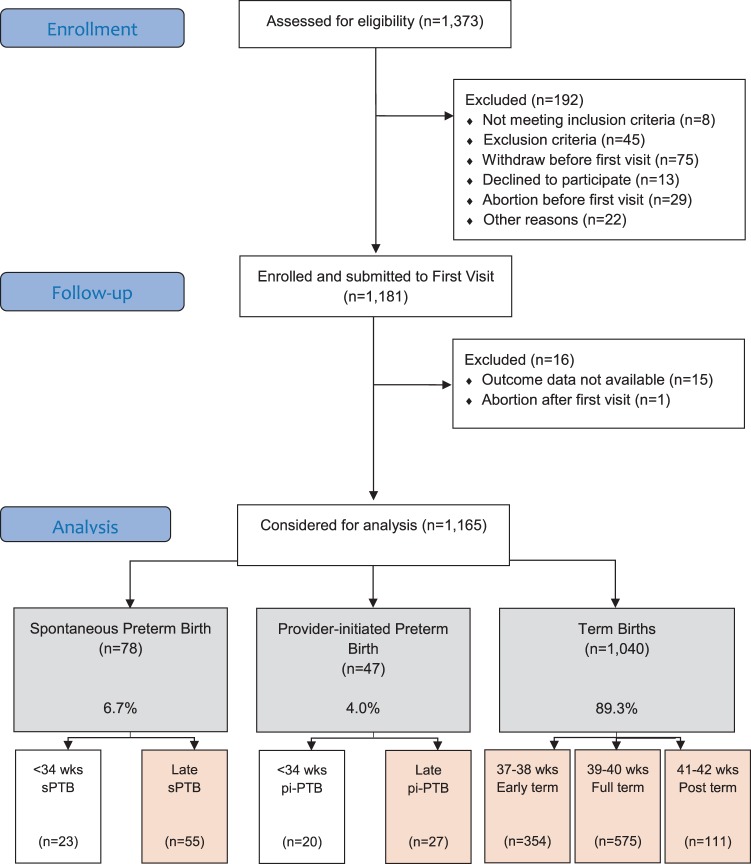
Preterm SAMBA Flowchart – Preterm birth subtypes’ analysis.

Preterm SAMBA study included 1,181 participants, from which 1,165 were followed and had outcome data available (Fig. 1). Preterm birth rate was 10.7% (n = 125). From the 78 cases of sPTB and 47 of pi-PTB, 55 (70.5%) and 27 (57.4%) were late preterm births. From the 1,040 term births, 354 (34.0%) were early term (37–38 weeks), 575 (55.3%) full term (39–40) and 111 (10.6%) post-term (41–42 weeks). Supplementary info (S1) shows a histogram of the distribution of gestational age (in days) at birth. None of the studied maternal and sociodemographic characteristics were different between sPTB, pi-PTB and term births (Table 1). Before the admission when the birth occurred, women who had sPTB had significantly more cerclage (RR 3.62, 95% CI [1.07–12.22]), pessary use (RR 5.55, 95% CI [3.17–9.71]), history of preterm labour or pPROM (RR 8.27, 95% CI [3.70–18.51]), use of antenatal steroids (RR 9.45, 95% CI [7.19–12.42]) or tocolysis (RR 6.27, 95% CI [2.73–14.42]) compared to women who had term birth (Table 2). Women who had pi-PTB had pessary and antenatal steroids use approximately 6 and 26 times more frequent, respectively, than women with term births.

**Table 1:** Maternal characteristics from women who had sPTB, pi-PTB and term births.

Characteristics	sPTB	pi-PTB	Term births	p-value*
**Region**				0.329
Northeast	34 (43.6%)	25 (53.2%)	506 (48.7%)	
South and Southeast	44 (56.4%)	22 (46.8%)	534 (51.3%)	
**Maternal age (years)**				0.137
≤19	18 (23.1%)	4 (8.5%)	269 (25.9%)	
20–34	54 (69.2%)	37 (78.7%)	705 (67.8%)	
≥35	6 (7.7%)	6 (12.8%)	66 (6.3%)	
**Ethnicity**				0.429
White	32 (41.0%)	14 (29.8%)	416 (40.0%)	
Non-white	46 (59%.0)	33 (70.2%)	624 (60.0%)	
**Marital status**				0.127
With partner	52 (66.7%)	39 (83.0%)	762 (73.3%)	
Without partner	26 (33.3%)	8 (17.0%)	278 (26.7%)	
**Maternal Occupation**				0.085
Paid work	41 (52.6%)	32 (68.1%)	512 (49.2%)	
Housewife	13 (16.7%)	6 (12.8%)	192 (18.5%)	
Not working	24 (30.7%)	9 (19.1%)	360 (32.2%)	
**Schooling (years)**				0.883
<12	52 (66.7%)	33 (70.2%)	706 (67.9%)	
≥12	26 (33.3%)	14 (29.8%)	334 (32.1%)	
**Annual Family Income (US$)**				0.519
Up to 12,000	43 (55.1)	31 (66.0%)	611 (58.8%)	
Above 12,001	35 (44.9%)	16 (34.0%)	429 (41.3%)	
**Source of prenatal care**				0.602
Entirely public	67 (85.9%)	42 (89.4%)	899 (86.4%)	
Private/insurance/mixed	11 (14.1%)	5 (10.6%)	141 (13.6%)	
**Total**	**78**	**47**	**1,040**	

* Chi-squared considering cluster-design effect.

**Table 2:** Antenatal and peripartum management characteristics of sPTB, pi-PTB and term births.

Characteristics	sPTB	RR (95%CI)	pi-PTB	RR (95%CI)	Term births
Use of vaginal progesterone*^a^					
None	62 (80.5%)	Ref.	42 (91.4%)	Ref.	987 (96%)
1 Trimester only	6 (7.8%)	3.76 [078–18.01]	2 (4.3%)	2.13 [0.24–18.96]	21 (2%)
1^st^, 2^nd^ and/or 3^rd^ trimesters	9 (11.7%)	5.08 [1.76–14.66]	2 (4.3%)	2.13 [0.42–10.74]	21 (2%)
**Cerclage**					
Yes	1 (1.3%)	**3.62 [1.07–12.22]**	0 (0%)	—	4 (0.3%)
No	77 (98.7%)	Ref.	47 (100%)	Ref.	1,161 (99.7%)
**Pessary**					
Yes	3 (3.8%)	**5.55 [3.17–9.71]**	2 (4.3%)	**6.86 [3.39–13.88]**	5 (0.5%)
No	75 (96.2%)	Ref.	45 (95.7%)	Ref.	1,035 (99.5%)
**History of preterm labor or pPROM**^**#**^					
Yes	24 (30.8%)	**8.27 [3.70–18.51]**	1 (2.1%)	0.67 [0.02–19.09]	33 (3.2%)
No	54 (69.2%)	Ref.	46 (97.9%)	Ref.	1,007 (96.8%)
**Antenatal Steroids**^**#a**^					
Yes	33 (50%)	**9.45 [7.19–12.42]**	31 (73.8%)	**26.53 [7.97–88.32]**	51 (6.3%)
No	33 (50%)	Ref.	11 (26.2%)	Ref.	761 (93.7%)
**Tocolysis**^**#**^					
Yes	8 (10.3%)	**6.27 [2.73–14.42]**	2 (4.3%)	3.41 [0.23–51.27]	12 (1.2%)
No	70 (89.7%)	Ref.	45 (95.7%)	Ref.	1,028 (98.8%)

^*^Initiated until 28 weeks. ^#^Before the admission when the birth occurred; childbirth did not occur during the admission due to preterm labor or pPROM, and women were discharged. Missing information for: ^a^13.

Supplementary info (S2) shows that the frequency of use of antenatal steroids were 65.2% and 29.6% in women who had sPTB <34 weeks and 34–36 weeks, respectively. In pi-PTB, it was 73.7% and 56.0% for <34 weeks and 34–36 weeks, respectively. Tocolysis was performed in 43.5% and 14% of cases of women who had a sPTB <34 weeks and 34–36 weeks (p-value 0.001), respectively.

Table 3 shows maternal and neonatal outcomes according to sPTB, pi-PTB and term birth cases. All neonatal adverse outcomes were significantly more frequent in preterm birth groups than in term. In addition, pi-PTB showed higher risk for almost all adverse neonatal outcomes compared to sPTB, including APO (RR 6.17, 95% CI [3.72–10.22] for sPTB and RR 25.39, 95% CI [10.08–63.96] for pi-PTB). pi-PTB cases had had 7 and 5 times more preeclampsia [95% CI 2.39–12.21] and small for gestational age neonates [95% CI 3.15–16.99], respectively, than women with term birth.

**Table 3:** Maternal and neonatal outcomes of sPTB and pi-PTB compared to full term births.

Characteristics	sPTB	RR [95% CI]	pi-PTB	RR [95% CI]	TermBirths
**Onset of labor**					
Spontaneous labour	65 (83.3%)	Ref.	0 (0%)	Ref.	617 (59.3%)
PROM + induction	9 (11.5%)	1.66 [0.69–3.95]	0 (0%)	—	48 (4.6%)
Induction intact membranes	0 (0%)	—	6 (12.8%)	—	184 (17.7%)
Elective C-section	4 (5.2%)	0.22 [0.03–1.50]	41 (87.2%)	—	191 (18.4%)
**Mode of delivery**					
Vaginal	57 (73.1%)	Ref.	4 (8.5%)	Ref.	556 (53.8%)
Intrapartum C-section	5 (6.4%)	0.65 [0.36–1.16]	1 (2.1%)	0.56 [0.02–20.32]	277 (23.8%)
Elective C-section	16 (20.5%)	0.23 [0.05–1.14]	42 (89.4%)	**21.73 [2.60–181.80]**	251 (24.1%)
**Length of maternal postpartum hospitalization**^**a**^					
1–3 days	62 (79.5%)	Ref.	28 (59.6%)	Ref.	951 (91.5%)
4–6 days	13 (16.7%)	**2.44 [1.12–5.33]**	13 (27.7%)	**5.22 [1.05–25.89**	74 (7.1%)
≥7 days	3 (3.8%)	2.88 [0.64–12.90]	6 (12.8%)	**10.49 [2.34–47.10]**	14 (1.3%)
**Preeclampsia**	2 (2.6%)	0.40 [0.07–2.18]	18 (38.3%)	**7.32 [3.15–16.99]**	67 (6.4%)
**HIP**^**b**^	12 (16.9%)	1.15 [0.39–3.44]	5 (12.5%)	0.83 [0.27–2.51]	133 (14.8%)
**Mean (±SD) birthweight (g)**^**c**^	2,253 ± 666.9	**1,002 [893.6–1111.0]**^**#**^	1,824 ± 845.7	**1,431 [921.9–1940.0]**_**#**_	3,255 ± 422.3
**Adequacy of birthweight to GA**^**c**^					
SGA (p < 10)	8 (10.2%)	0.94 [0.20–4.49]	45 (44.7%)	**5.40 [2.39–12.21]**	117 (11.4%)
AGA (p10–90)	58 (74.4%)	Ref.	23 (48.9%)	Ref.	793 (77.2%)
LGA (p > 90)	12 (15.4%)	1.36 [0.48–3.86]	3 (6.4%)	0.89 [0.14–5.65]	117 (11.4%)
**Fetal death**	1 (1.3%)	**14.51 [10.63–19.79]**	2 (4.3%)	**24.11 [18.88–30.79]**	0 (0%)
**Neonatal death**	2 (2.6%)	**9.90 [5.08–19.32]**	5 (10.6%)	**22.48 [10.99–45.99]**	1 (0.1%)
**Apgar score – 5**^**th**^ **minute** < **7**^**d**^	5 (6.8%)	**5.47 [2.46–12.15]**	5 (11.1%)	**9.04 [3.51–23.31]**	9 (0.9%)
**Need of intubation after birth**^**e**^	12 (15.4%)	**12.42 [8.85–17.43]**	10 (22.2%)	**21.67 [11.79–39.85]**	4 (0.4%)
**NICU admission**	40(51.3%)	**7.54 [3.65–15.58]**	36 (76.6%)	**23.47 [16.13–34.16]**	97 (9.3%)
**Phototherapy for jaundice**^**e**^	47 (61.0%)	**7.08 [3.68–13.60]**	31 (68.9%)	**10.68 [5.66–20.14]**	154 (14.9%)
**Length of NICU stay (days)**					
1–3 days	4 (10.0%)	Ref.	8 (22.2%)	Ref.	59 (60.8%)
4–6 days	5 (12.5%)	3.58 [0.54–23.81]	3 (8.3%)	1.26 [0.13–12.34]	17 (17.5%)
≥7 days	31 (77.5%)	**9.39 [1.73–51.06]**	25 (69.4%)	**4.55 [1.39–14.92]**	21 (21.6%)
**Neonatal sepsis**^**f**^	14 (18.4%)	**7.17 [4.54–11.33]**	10 (21.7%)	**9.76 [3.88–24.54]**	20 (1.9%)
APO* ^**g**^ **[95%CI]**	37 (50.0% [38.0–62.0%])	**7.17 [3.16–16.28]**	38 (80.9% [63.0–91.3%])	**29.13 [18.07–46.98]**	92 (9.4% [3.1–25.4%]}
**Total**	**78**		**47**		**1,040**

Missing information for: ^a^1; ^b^157; ^c^13; ^d^65; ^e^11; ^f^5; ^g^64. ^#^WMD, weighted mean difference [95% CI]. *APO: NICU stay >7 days or intubation at birth or Apgar score <7 at 5 minutes or fetal/neonatal death or discharge home on oxygen or neonatal sepsis or cyanosis or hypoglycaemia or birth asphyxia or respiratory distress or mechanical ventilation.

Table 4 shows maternal and neonatal outcomes for late sPTB, late pi-PTB, and early and full-term birth categories. Longer maternal postpartum hospitalization, NICU admission, phototherapy for jaundice and APO were more frequent in late sPTB and pi-PTB groups than full term birth. Women who had preeclampsia were more frequent in late pi-PTB (RR 7.5 [2.48–22.67]) and early term birth cases (RR 1.7 [1.36–2.13]). There were no cases of fetal death in late sPTB, late pi-PTB, and term birth groups (data not shown). There were few cases of neonatal death (1 early term) and need for intubation after birth (1 late pi-PTB, 3 early term and 1 full term).

**Table 4:** Maternal and neonatal outcomes of late preterm birth and early and post term compared to full term births.

Characteristics	Late sPTB34–36 wks	RR (95% CI)sPTB vs full term	Late pi-PTB34–36 wks	RR (95% CI)pi-PTB vs full term	Early term37–38 wks	RR (95% CI)Early term vs full term	Full term39–40 wks	Post term41–42 wks	RR (95% CI)Post term vs full term
**Onset of labor**									
Spontaneous labour	42 (76.4%)	Ref.	0 (0%)	Ref.	189 (53.4%)	Ref.	375 (65.2%)	53 (47.7%)	Ref.
PROM + induction	9 (16.4%)	**3.08 [1.45–6.56]**	0 (0%)	—	24 (6.8%)	**1.63 [1.24–2.14]**	20 (3.5%)	4 (3.6%)	1.35 [0.66–2.74]
Induction intact membranes	0 (0%)	—	4 (6.8%)	—	65 (18.4%)	1.32 [0.73–2.40]	82 (14.3%)	37 (33.3%)	2.51 [0.99–6.36]
Elective C-section	4 (7.2%)	0.39 [0.06–2.50]	23 (85.2%)	—	76 (21.4%)	1.30 [0.81–2.11]	98 (17.0%)	17 (15.4%)	1.19 [0.41–3.43]
**Mode of delivery**									
Vaginal	39 (70.9%)	Ref.	2 (7.4%)	Ref.	180 (50.8%)	Ref.	330 (57.3%)	49 (44.2%)	Ref.
Intrapartum C-section	11 (20.0%)	0.73 [0.30–1.77]	1 (3.7%)	1.25 [0.02–74.28]	81 (22.9%)	1.08 [0.82–1.41]	132 (23.0%)	38 (34.2%)	**1.73 [1.25–2.40]**
Elective C-section	5 (9.1%)	0.40 [0.09–1.72]	24 (88.9%)	**29.08 [1.34–630.29]**	93 (26.3%)	1.28 [0.91–1.79]	113 (19.7%)	24 (21.6%)	1.35 [0.46–4.01]
**Length of maternal postpartum hospitalization**^**a**^									
1–3 days	40 (72.7%)	Ref.	16 (59.3%)	Ref.	318 (90.1%)	Ref.	534 (92.9%)	99 (89.2%)	Ref.
4–6 days	12 (21.8%)	**3.91 [1.57–9.77]**	9 (33.3%)	7.55 [0.86–65.93]	32 (9.1%)	1.34 [0.68–2.64]	32 (5.6%)	10 (9.0%)	1.52 [0.41–5.61]
≥7 days	3 (5.5%)	3.59 [0.78–16.40]	2 (7.4%)	**6.25 [1.08–36.13]**	3 (0.8%)	0.67 [0.10–4.46]	9 (1.5%)	2 (1.8%)	1.16 [0.19–7.12]
**Preeclampsia**	1 (1.8%)	0.45 [0.05–3.69]	8 (29.6%)	**7.50 [2.48–22.67]**	39 (11.0%)	**1.70 [1.36–2.13]**	24 (4.2%)	4 (3.6%)	0.88 [0.13–6.10]
HIP*^b^	9 (18.8%)	1.23 [0.33–4.66]	3 (13.0%)	0.83 [0.35–1.97]	52 (16.4%)	1.04 [0.73–1.47]	76 (15.5%)	5 (5.7%)	0.37 [0.08–1.81]
**Mean (SD) birthweight (g)**^**c**^	2,533 ± 457	**793.8 [649.7–938.0]#**	2,403 ± 520	**924.4 [622.4–1,226.4]#**	3,059 ± 390	**268.2 [214.7–321.7]#**	3,327 ± 388	3,508 ± 438	**−180.9 [(−269.3)- (−92.6)]#**
**Adequacy of birthweight to GA**^**d**^									
SGA (p < 10)	6 (10.9%)	1.11 [0.18–6.74]	7 (25.9%)	**2.99 [1.08–8.32]**	43 (12.2%)	1.16 [0.82–1.63]	57 (10.1%)	17 (15.3%)	1.52 [0.73–3.14]
AGA (p10–90)	42 (76.4%)	Ref.	17 (63.0%)	Ref.	265 (75.1%)	Ref.	448 (79.6%)	80 (72.1%)	Ref.
LGA (p > 90)	7 (12.7%)	1.26 [0.36–4.36]	3 (11.1%)	1.35 [0.31–5.85]	45 (12.7%)	1.18 [0.81–1.70]	58 (10.3%)	14 (12.6%)	1.28 [0.47–3.54]
**Neonatal death**	0 (0%)	—	0 (0%)	—	1 (0.3%)	**2.63 [2.25–3.08]**	0 (0%)	0 (0%)	—
**Apgar score – 5**^**th**^ **minute** < **7**^**e**^	1 (2.0%)	1.95 [0.15–24.90]	2 (7.4%)	6.40 [0.67–60.70]	4 (1.2%)	1.16 [0.46–2.97]	5 (0.9%)	0 (0%)	—
**Need of intubation after birth**^**f**^	0 (0%)	—	1 (3.7%)	**11.46 [6.60–19.91]**	3 (0.9%)	**1.99 [1.07–3.69]**	1 (0.2%)	0 (0%)	—
**NICU admission**	19 (34.5%)	**4.16 [1.87–9.23]**	18 (66.7%)	**15.20 [10.80–21.39]**	35 (9.9%)	1.06 [0.89–1.27]	52 (9.0%)	10 (9.0%)	1.00 [0.84–1.18]
**Phototherapy for jaundice**^**g**^	29 (52.7%)	**4.85 [2.90–8.13]**	17 (63.0%)	**7.98 [2.85–22.39]**	54 (15.3%)	1.00 [0.87–1.15]	88 (15.4%)	12 (11.0%)	0.72 [0.33–1.58]
**Length of neonatal admission (days)**									
1–3 days	3 (15.8%)	Ref.	5 (27.8%)	Ref.	22 (62.9%)	Ref.	31 (59.6%)	6 (60.0%)	Ref.
4–6 days	4 (21.1%)	2.83 [0.22–36.24]	2 (11.1%)	1.03 [0.26–4.15]	3 (8.5%)	0.48 [0.08–3.03]	12 (23.1%)	2 (20.0%)	0.88 [0.04–20.09]
≥7 days	12 (63.1%)	6.48 [1.00–42.05]	11 (61.1%)	**3.96 [1.29–12.13]**	10 (28.6%)	1.27 [0.94–1.71]	9 (17.3%)	2 (20.0%)	1.12 [0.10–12.30]
**Neonatal sepses**^**h**^	3 (5.6%)	**2.58 [1.71–3.90]**	1 (3.7%)	1.89 [0.18–19.88]	6 (1.7%)	0.93 [0.35–2.42]	11 (1.9%)	3 (2.7%)	1.33 [0.32–5.62]
APO* [95%CI]	15 (29.4% [17.8–44.5%])	**3.37 [1.45–7.84]**	18 (66.7% [44.5–83.3%])	**14.65 [9.50–22.57]**	34 (10.2% [3.3–27.6%])	1.07 [0.90–1.26]	50 (9.3% [3.3–23.7%])	8 (7.4% [1.4–31.1])	0.81 [0.46–1.43]
**Total**	**55**		**27**		**354**		**575**	**111**	

Missing information for: ^a^1; ^b^154; ^c^1; ^d^13; ^e^62; ^f^9; ^g^8; ^h^3; ^i^64. ^*^Hyperglycemia in pregnancy. #WMD, weighted mean difference [95% CI]. *APO (Any Adverse Perinatal outcome): NICU stay >7 days or intubation at birth or Apgar score <7 at 5 minutes or fetal/neonatal death or discharge home on oxygen or neonatal sepsis or cyanosis or hypoglycaemia or birth asphyxia or respiratory distress or mechanical ventilation.

Elective C-section was much more frequent in pi-PTB (89.4%) and late pi-PTB (88.9%) than in overall term births (24.1%) or full-term birth (19.7%). Overall C-section (including elective and intrapartum) were performed in 91.5% of pi-PTB cases, 47.9% of term births and 26.9% of spontaneous preterm birth.

Table 5 shows that pi-PTB (RR_adj_ 8.12, 95% CI [2.54–25.93], p-value 0.007), maternal WGR (RR_adj_ 2.04, 95% CI [1.23–3.38], p-value 0.018) and women from the northeast participating centres (RR_adj_ 2.35, 95% CI [1.11–4.95], p-value 0.034) were independently associated with any adverse perinatal outcomes.

**Table 5:** Factors independently associated with APO* in preterm newborns: multiple analyses by Poisson regression [n = 837].

Variables	RR_adj_	95%CI	p-value
Pi-PTB	**8.12**	**2.54–25.93**	**0.007**
Weight gain rate per week 20–27 weeks <p10	**2.04**	**1.23–3.38**	**0.018**
Region (Northeast)	**2.35**	**1.11–4.95**	**0.034**

Variables included in the model: Region; ethnicity; annual family income; source of prenatal care; smoking status; previous maternal condition; cervical length from 18 to 24 weeks <25 mm; weight gain rate per week 20–27 weeks <Q1; weight gain rate per week 20–27 weeks <Q2; weight gain rate per week 20–27 weeks <p10; weight gain rate per week 20–27 weeks >p90; sPTB; pi-PTB; gestational age at birth; Preeclampsia; Hyperglycaemia in pregnancy. *APO: NICU stay >7 days or intubation at birth or Apgar score <7 at 5 minutes or fetal/neonatal death or discharge home on oxygen or neonatal sepsis or cyanosis or hypoglycaemia or birth asphyxia or respiratory distress or mechanical ventilation.

## Discussion

Despite being considered at term, early term neonates present poorer adverse outcomes when compared to full term and caution with “liberalization” in pregnancy resolution in this pregnancy interval should be taken^15,16^. Similarly to late preterm, early term neonates are associated with higher prevalence of NICU admission, need for oxygen therapy, hypoglycaemia, neonatal mortality and other neonatal morbidities when compared to full term neonates^15^. Neonatal mortality is around 2.3 times higher in 37 weeks compared to 39 weeks neonates^6^. ACOG reinforced the importance of delaying, when possible, the elective resolution of pregnancy to after 39 weeks, rather than intervening at 37 or 38 weeks^7^. In 2016, the Brazilian Federal Council of Medicine, an independent agency responsible for professional regulation of medical doctors, established a normative resolution establishing that elective C-section due to patient request should only be performed after 39 weeks of gestation^17^. The concept of “too much, too soon” and “too little, too late” can be properly applied in this discussion^18^. Adequate management of obstetric interventions (induction of labour, C-section, recognition of maternal/fetal complication, etc.) during late preterm and early term is a complex challenge in the Brazilian context, where there are disparities in access to intensive maternal and neonatal care units^10,19,20^ and high rates of preventable severe maternal morbidities related to preeclampsia^21,22^. Brazil shows to be a country where over-medicalization and misuse of obstetric interventions walk together with a lack of well-trained multidisciplinary team of health care providers and insufficient equipment and resources.

The use of tertiary preventive strategies such as antenatal corticosteroids (ACS) and tocolysis does not prevent preterm birth, but may improve associated neonatal outcomes^23–26^. Around 65% of sPTB before 34 weeks and 30% of late sPTB used ACS. The EMIP study, a multicentre cross-sectional study in 20 referral maternities in Brazil, showed that ACS was used in 54.0% of sPTB before 34 weeks and in 14.0% of late sPTB^27^. In accordance with the evidence-based recommendation for using ACS between 34 and 36 weeks raised by ALPS study^28^ in 2016 and by 2017 Cochrane Systematic review^23^, an increase in the use of ACS can be observed between both Brazilian studies, EMIP (2011–2012) and Preterm SAMBA (2015–2018). In our study, half of women who had sPTB and around 70% who had pi-PTB had used ACS before the admission when birth occurred (Supplementary Info - Table S1). The effect of repeated doses and the benefits of ACS in low-resourced settings remain controversial. The WHO reported that there is a need for further investigation of ACS effects in low-resource settings, where the estimate of gestational age may not be accurate enough^29^. According to the Birth in Brazil study, information of an early ultrasound was available for only 44.5% of women^30^.

Women who had a pi-PTB were independently associated with perinatal adverse outcomes. The EMIP study, a cross-sectional study that carried out surveillance of preterm births in 20 referral obstetric centres in Brazil, showed that hypertensive disorders motivated around 90% of pi-PTB due to maternal conditions^31^. This study also showed that the neonatal mortality of extreme and late pi-PTB neonates before discharge were 200 and 6 times higher, respectively, in comparison to term neonates. A pi-PTB is a medical intervention to improve maternal and perinatal health condition, but it requires an evidenced-based decision-making process in order to avoid unnecessary prematurity and, consequently, more adverse neonatal outcomes. The HYPITAT II clinical trial showed that labour induction between 34 and 37 weeks of gestation in women with hypertensive disorders reduces adverse maternal complication^32^. Respiratory distress, however, were more frequent in the intervention group (RR 3.3, 95% CI [1.4–8.2; p = 0.005]). pi-PTB is associated with severe maternal morbidity and the decision-making process requires optimal resources to assure timely interventions, since any delay is also associated with more severe maternal outcomes^33,34^. The evidence that maternal morbidity leads to such adverse perinatal outcomes related to preterm birth reinforces the need for monitoring the occurrence of maternal morbidity, maternal near miss and the effects of related interventions to reduce both maternal and perinatal adverse outcomes.

Maternal WGR between 20 and 27 weeks below the tenth percentile was independently associated with APO. We acknowledge the fact that the absence of initial BMI is a great limitation for further interpretations. We did not use a standard definition for adequacy of weight gain as maternal early/pre-pregnancy body mass index was not available. Therefore, we addressed perinatal outcomes according to the different weight gain percentile and quintiles. We acknowledge the fact that 43% of women in Preterm SAMBA study were overweight or obese (data not shown) and only 39% were normal weight at 20 weeks according to the Atalah and cols´ reference ranges^35^. The recommendations of gestational weight gain from the Institute of Medicine - 2009 remains controversial as it did not take into account different populations and the effect of weight gain to the different preterm birth subtypes^36^. Poor maternal weight gain during pregnancy has been associated with adverse perinatal outcomes such as small for gestational age and preterm birth. A study evaluating more than 500,000 normal weight women and 230,000 overweight women showed that deviations of weight gain are associated with small for gestational age^37^. This association depends on how the exposure variable will be applied (total weight, rate of weight gain or adequacy according to IOM recommendation). Further studies evaluating the Brazilian population is required to better explore the risks for maternal and perinatal outcomes.

Although our findings are not innovative, we acknowledge the fact that it is a prospective low-risk nulliparous women cohort where, in theory, are expected to have low frequency of maternal and perinatal complications. Nevertheless, we have the opportunity to report important indicators as the frequency of perinatal outcomes for this population, that can be used to plan and monitor strategies to ameliorate maternal and perinatal health care. For instance, almost 50% of women who delivered at term had a C-section. We did not evaluate the indication for elective or intrapartum C-section, but such high rates in this population (nulliparous women) requires a careful attention. There were only 12.8% of induction in pi-PTB cases. C-section can be a life-saving procedure for both mother and fetus, and a balance between risk and benefits might be context specific^38^. In Brazil, C-section rate is certainly unbalanced.

A Cochrane systematic review showed that there are a plenty of non-clinical interventions to reduce unnecessary C-sections, including education programmes for women, training programmes for professionals, implementation of midwifery-labourist care and clinical practice guidelines to better support its indication^39^. The use of an institutional standardized classification of C-section to monitor its incidence is also a highly recommended approach^40,41^. According to a systematic review, there are at least 27 classifications based on different factors including women´s characteristics, degree of urgency and indications^42^. The Robson´s ten-group classification, based on obstetric characteristics as parity, previous C-section, preterm birth, onset of labour, fetal presentation and the number of fetuses, seems to be the most adequate;^40,42^ it can be easily implemented and used for longitudinal monitoring. A limitation is that the tenth group, comprised of all preterm birth cases, does not differentiate cases according to other obstetric characteristics^41^. Nulliparous women is a priority group when avoiding unnecessary C-sections due to its consequences to the women´s reproductive and general health^43^.

Participants from the Northeast centres were independently associated with APO. The definition of APO was based on some complications that require NICU; it can be used as a proxy of adverse outcomes or as an indicator of birth and neonatal care, reflecting the unmet need for allocation of financial and human resources. Our study was conducted in five referral obstetric facilities placed in four states of Brazil. The HDI in 2010 were 0.783 and 0.746 from the South/Southeast states, and 0.673 and 0.682 from the Northeast states^44^. According to a population-based study conducted in 2006 in Brazil, the proportion of women with inadequate prenatal care, low schooling and low income is higher in the Northeast when compared to the South/Southeast^19^. A more recent study, hospital-based comprising almost 24,000 women in Brazil, reinforced the existence of huge disparities in the different regions of Brazil^10,20^. The Northeast lacks of adequate prenatal care and maternities´ human and equipment resources when compared to South/Southeast regions. The nationwide hospital-based Birth in Brazil study addressed the hospital structure for birth and neonatal care in Brazil^45^. It demonstrated that 50% of obstetric risks were born in maternity units without a NICU. This proportion raised to 60% when in the North and Northeast regions. Also, only 10% of newborns at obstetric risk were born in public hospitals with a NICU whose structure was classified as appropriate^45^. Secondary analyses of the World Health Organization Multicountry Survey on Maternal and Newborn Health showed that the provision of care and maternal and perinatal outcomes vary according to the human development index (HDI)^4^. As the HDI increase, the proportion of adverse perinatal outcomes seems to decrease and pi-PTB, on the contrary, to increase. Also, the accessibility to a preterm resolution of pregnancy when required is limited for younger women and for those with lower schooling^4^.

There are strengths and limitations in our study. Early term neonates had 2.6 times more risk for neonatal death and pi-PTB and early term neonates had approximately 11 and 2 times more risk, respectively, for need of intubation. Although remarkable, these findings should be interpreted with caution due to the low number of cases in each group. We only evaluated perinatal and short-term neonatal outcomes, before neonate’s and woman’s discharge. New hospital admissions or complications were not evaluated. Comprehensive multicentre studies evaluating long-term outcomes of preterm birth in low- and middle-income countries are of urge importance. Brazil is a huge country with regional and private/public system inequalities in maternal, perinatal an infant health care^10,20^. Low resource settings have the highest rates of maternal and neonatal morbidity and mortality^46–49^, and are the neediest places where improving quality of antenatal care, investments in preterm birth research and implementing maternal and perinatal high evidence-based care will impact the most.

## Supplementary information

Supplementary information.

## Data Availability

The dataset used and analysed during the current study is available from the corresponding author on reasonable request.
