# Supplementary material for: Perinatal outcomes from preterm and early term births in a multicenter cohort of low risk nulliparous women
Source: Sci Rep. 2020 May 22;10:8508. doi: 10.1038/s41598-020-65022-z (PMC7244568; doi:10.1038/s41598-020-65022-z)
Supplement: Supplementary file 1 — Supplementary information. [file 41598_2020_65022_MOESM1_ESM.docx]

*CLINICAL ARTICLE*

**Perinatal outcomes from preterm and early term births in a multicenter cohort of low risk nulliparous women**

Renato T Souza, Maria L Costa, Jussara Mayrink, Francisco E. Feitosa, Edilberto A Rocha Filho, Débora F Leite, Janete Vettorazzi, Iracema M Calderon, Maria H Sousa, Renato Passini Jr, Philip N Baker, Louise Kenny, Jose G. Cecatti, for the Preterm SAMBA study group

**S2. Histogram of gestational age at birth in the Preterm SAMBA cohort**

**
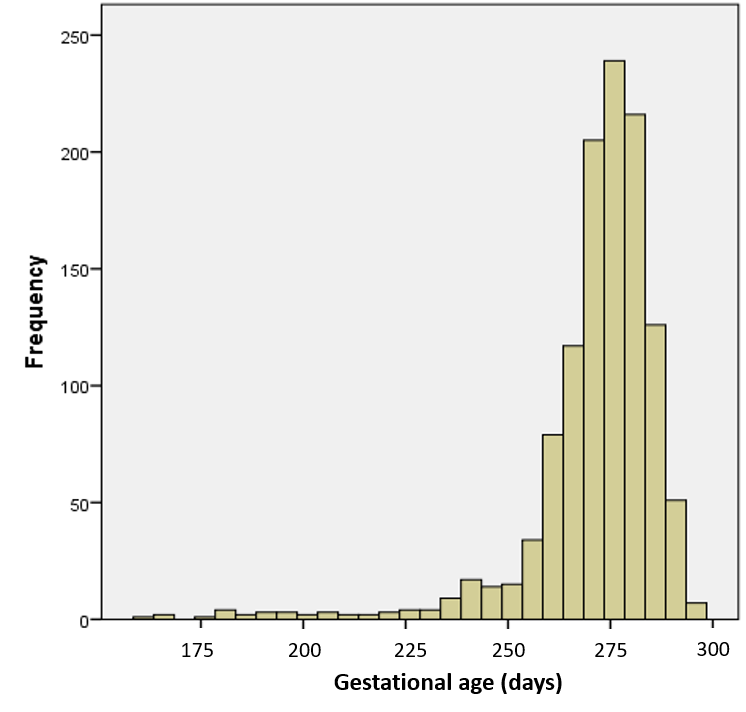
**

**S1. Peripartum management characteristics according to spontaneous and provider-initiated preterm birth categories**

| **Characteristics** | **sPTB** | |  | **pi-PTB** | |  |
| --- | --- | --- | --- | --- | --- | --- |
|  | **<34w** | **34-36w** | **p-value** | **<34w** | **34-36w** | **p-value** |
| **Tocolysis*^a^** |  |  | **0.001** |  |  | - |
| Yes | 10 (43.5%) | 7 (14.0%) |  | 0 (0%) | 0 (0%) |  |
| No | 13 (56.5%) | 43 (86.0%) |  | 15 (100%) | 17 (100%) |  |
| **Antenatal Steroids*^b^** |  |  | **0.033** |  |  | 0.378 |
| Yes | 15 (65.2%) | 16 (29.6%) |  | 14 (73.7%) | 14 (56.0%) |  |
| No | 8 (34.8%) | 37 (70.4%) |  | 5 (26.3%) | 11 (44.0%) |  |

*****during admission when birth occurred. Missing information for sPTB and pi-PTB, respectively: a) 5 and 15; b) 2 and 3.
